# Case of Glandular Tumor of Neck

**Published:** 1859-11

**Authors:** James Cody

**Affiliations:** Watertown. Wis.


ARTICLE V.

### CASE OF GLANDULAR TUMOR OF NECK.

SUCCESSFUL OPERATION.

BY JAMES CODY, M.D., OF WATERTOWN, WIS.

July 14th, 1859.—Mr. Truman Gates, aged 34 years, of nervous sanguine temperament; has always enjoyed good health. Five years ago he first noticed a small round tumor, just under the left jaw-bone, midway between the angle of the jaw and symphysis. From that time it has steadily increased, without pain or any other inconvenience, until four months since, when it was noticed to increase rapidly, causing much inconvenience from its mechanical pressure on the surrounding parts.

*Present condition and appearance of patient.*—The left side of the neck presents a large globular swelling, as large or larger than the fist. It commences half an inch below the ear, and extends nearly an inch beyond and to the right side of the symphysis; its upper edge rests on the jaw-bone; its lower edge nearly describes a semi-circle; covering the side of the neck, midway to the clavicle; pressing the larynx and upper part of the trachea to the right of the median line full half an inch or more; to the left it apparently pressed the sterno-mastoid muscle out or rather to the left.

On passing the finger into the mouth and under the tongue, the tumor can easily be felt through the floor of the mouth and pressing against the root of the tongue. He complains of a difficulty of swallowing; his speech is affected; the voice has a peculiar indescribable sound; some pain in head; face somewhat flushed, and a number of turgid veins are seen passing over the swelling. He says that the pressure of the tumor gives him very unpleasant feelings, and wishes for its removal, as he is conscious it will soon terminate his existence.

The operation was performed in the following manner: Having administered chloroform, an incision was made, extending over the long diameter of the swelling, commencing, perhaps, one inch below the angle of the jaw, and carrying it seven inches, or about opposite cricoid cartilage. This incision was carried through the integument and down to the platysma-myoides muscle. This muscle, with the deep cervical fascia, was now cut through, when the tumor was brought into view. The rest of the dissection was for the most part conducted with the fingers and handle of the scalpel, only occasionally using the edge of the knife to divide bands of cellular tissue. The tumor dipped deep down by the side of the larynx; also mounted up by the inner side of the jaw-bone, being most prominent opposite the molar teeth.

After the tumor was turned out, it was found to be attached by a pedicle half an inch or more in circumference. For fear and to avoid hemorrhage, the pedicle was ligated and the tumor removed.

The tumor was bi-lobed or kidney-shaped, dense and hard to the feel, except one spot, which was soft and seemed to contain a fluid. It measured nine inches in its longest diameter and seven and three-fourths in circumference.

In a few hours after the operation he became very restless; some muscular twitching was noticed; the neck and left side of the face became swollen, but not painful; pulse considerably accelerated. A dose of morphine was administered, and ice water applied to the swelling; after which he went to sleep, and on waking up felt very comfortable. In the evening one of the sutures was taken out and all the adhesive straps removed. Continued ice water; also gave tart. emet. in solution, quarter of a grain every two hours.

July 15th, 8 a.m. Rested well during night; pulse 90, rather hard; face and neck swollen on left side; continued sol. tart. ant.; apply sol. mur. ammoniæ to swelling; drink, lemonade and cream tartar.

16th, 8 a.m. Spent a comfortable day yesterday; had two dejections; no difficulty in swallowing; voice natural; some discharge from wound; slept well last night; continue mur. ammoniæ; take sol. nit. pot. six gr. every three hours in flaxseed water; urine scanty and high colored.

17th, 8 a.m. Reports himself as comfortable; swelling not decreasing much; ordered flaxseed poultice to face and neck; continue nit. pot., also take cream tartar.

18th, 8 a. m. Is doing well; considerable discharge from wound; continue poultice, etc.

19th. The same as yesterday.

20th. The swelling is fast decreasing; large discharge of pus.

21st, 8 a.m. Swelling decreasing; complains of soreness of throat and difficulty of swallowing; ordered gargle of nit. argent. gr. ij. to ounce of water.

22nd, 8 a.m. Soreness of throat less to-day; continue gargle.

23rd, 8 a.m. The same as yesterday.

24th, 8 a.m. Is not so well to-day; complains of more pain in throat—a sensation of filling up; tongue furred; breath of fetid odour; is to take castor oil ʒij., oil terebinth. ʒi., calc. mag. ℈ij.; after operation and to-night is to take Doveri pulv. et bi-carb. soda, aa. gr. v. every three hours.

25th, 8 a.m. Reports himself as feeling quite well to-day; swallows well; no pain or soreness of throat. He thinks an abscess broke inside the throat last night, as he spat up matter and experienced immediate relief. Cathartic operated three times in the afternoon yesterday. He took but two powders last night; is to suspend medicine; swelling fast decreasing; wound three-fourths healed; apply simple cerate to wound; flaxseed, mixed up in sal. mur. ammon., to swelling above the wound.

26th, 8 a.m. Considers himself well; swelling nearly all gone.

27th, 8 a.m. Doing well; was about town all day yesterday; starts for home, twenty miles distant, to-day.

27th. Mr. Gates called to-day and reported himself as well. There is no swelling or enlargement whatever of the left side of neck and face; on inspection, no difference of the two sides of the face can be detected.

